# Effectiveness of medical taping concept in primary dysmenorrhoea: a two-armed randomized trial

**DOI:** 10.1038/srep16671

**Published:** 2015-11-13

**Authors:** María Isabel Tomás-Rodríguez, Antonio Palazón-Bru, Damian Robert James Martínez-St. John, José Vicente Toledo-Marhuenda, María del Rosario Asensio-García, Vicente Francisco Gil-Guillén

**Affiliations:** 1 Department of Pathology and Surgery, Miguel Hernández University, San Juan de Alicante, Alicante, Spain; 2 Department of Clinical Medicine, Miguel Hernández University, San Juan de Alicante, Alicante, Spain; 3 Physiotherapy Office, Da Vinci Clinic, Santa Faz, Alicante, Spain; 4 Rehabilitation Unit, University Hospital, San Juan de Alicante, Alicante, Spain

## Abstract

In 2014, we assessed the effectiveness of Medical Taping Concept (MTC) in Primary Dysmenorrhoea (PD) with a single-blind, two-armed clinical trial (NCT02114723, ClinicalTrials.gov) with a follow-up of 4 menstrual cycles (pre-intervention: 2 months; post-intervention: 2 months) in a sample formed by 129 Spanish women aged 18–30 years with PD. We had two groups: intervention group (75), MTC covering T-11 and T-12 dermatomes; control group (54), another taping in both greater trochanter areas. Our main outcome measures were: pre-intervention and post-intervention increase in pain difference measured 2 hours after commencement (*2-h pain* — *0-h pain*); difference between the number of tablets ingested post-intervention and pre-intervention; and associated symptoms in post-intervention (fatigue, vomiting, diarrhoea, nausea and others). Pain was assessed in: abdomen, legs, head and lower back. We found significant differences (p < 0.05) for number of tablets, abdominal and leg pain. In conclusion, the intervention group had less abdominal and leg pain when pharmacological therapy was not started. Furthermore, the intervention resulted in a lower intake of tablets. Nevertheless, more studies are needed to corroborate our results and to analyze the MTC effectiveness if women do not take any tablets during the entire menstrual period.

Primary dysmenorrhoea (PD) is one of the most frequent gynaecological alterations, causing work and academic absence in women, due to its monthly and disabling nature^1^. This type of pain is very relevant due to its periodic nature, intensity and because it’s incapacitating, but few women seek medical help^2^.

Many drugs have been suggested to treat PD. In spite of their effectiveness it has been necessary to constantly try to find alternative therapies for patients who cannot benefit from traditional pharmacological treatments because of the adverse effects. These therapies include vegetarian diets, dietary supplements, medicinal herbs, acupuncture, and even, in extreme cases, surgical treatment^3^.

Another alternative has been physiotherapy, as certain techniques have been used to try to help these patients. The most frequent have been: the use of heat, massage, trans-cutaneous electrical stimulation, short wave, directed physical exercise and vertebral manipulations^4^,^5^,^6^,^7^,^8^,^9^,^10^,^11^,^12^,^13^,^14^,^15^,^16^,^17^,^18^,^19^,^20^,^21^,^22^,^23^. However, because of the great number of available techniques in this area, known for the lack of secondary effects, requires the larger studies to be carried out with the aim of validating different and novel therapeutic procedures. Despite the apparent efficacy of these procedures and their frequent use, there is a lack of solid scientific evidence, like in the case of kinesiotaping or medical taping concept (MTC). This could be a potential technique to reduce this type of pain, as it produces sensory tactile impulses on the skin that can block or reduce the arrival of pain sensations to the brain^24^.

In our literature search, we only found one study published in the English language, although we did find one published in Dutch, other in Korean and another in Spanish, but these were published in low impact international journals, which analyzed the effectiveness of MTC in menstrual pain and pre-menstrual syndrome in a sample formed by 34 women^25^. This fact, justifies the need of carrying on making controlled clinical interventions which will increase the scientific evidence in this field. Therefore, we carried out a two-armed randomized clinical trial with four months of follow- up (4 menstrual cycles) which will fill the gap of the MTC effectiveness.

## Methods

### Study population

Female students of the School of Medicine from the Universidad Miguel Hernández of Elche (Spain) who suffer from PD.

### Study design and participants

The study was designed as a randomized, single-blind, two armed clinical trial with a 4-month follow-up (4 menstrual cycles). The assignment ratio was 1:1. The study was conducted in two phases, each measuring two menstrual cycles: 1) pre-intervention: two menstrual cycles before the intervention and 2) post-intervention: 2 menstrual cycles after the intervention.

Inclusion criteria for participation in the study were: women suffering from PD at the School of Medicine from the Universidad Miguel Hernández of Elche, aged between 18 and 30, to have undergone a general gynaecological revision in the past 18 months, to not have been diagnosed with secondary dysmenorrhoea by her gynaecologist, to have regular menstrual cycles (typical range between 21 and 35 days)^26^, to not have an inter-uterine contraceptive device or to be on oral contraceptive pills and to be nulliparous. PD was defined as presenting either grade 2 (menstruation with moderate pain with influence on daily activity and use of analgesics for pain relief) or grade 3 (menstruation with severe pain with significant limitation to daily activity, ineffective use of analgesics, and such symptoms as headache, tenderness, nausea, vomiting, and diarrhoea) on the Andersch and Milsom scale^27^. Exclusion criteria were: to have been diagnosed with a severe comorbid disorder^28^, to have undergone surgery during the study, to have skin lesions on the abdominal area (scars, erosions or cysts) and to have suffered a traumatic event during the study.

To recruit the sample of participants we obtained permission from professors teaching different courses at the School of Medicine to access the students in one of their classes. After a short presentation about the study’s aims and having obtained the participant’s cooperation, a first questionnaire was handed to all students reporting menstrual pain, to detect how many of them met all the inclusion and none of the exclusion criteria. Once this process was completed (recruitment), they were contacted over the phone to set up a meeting to explain the study and hand over the questionnaires, which they had to complete during the following two menstrual cycles (*pre-intervention*). After the questionnaires were completed, instructions were given about how to use the techniques (intervention or control) and all materials were handed over as well as new questionnaires. After the handover (*post-intervention*), the following two menstrual cycles were taken into account. Once the pain had started, the participant used the technique that had been assigned to her and measured the pain during that period using the questionnaires. They were also instructed that for the first two hours after applying the technique they were not to use any medication. If pain became unbearable, they were allowed to use their normal medication. Group allocation was decided using random numbers which were obtained with Epidat 3.1. Participants had no previous knowledge of the existence of the technique they were not assigned. Therefore, they knew two techniques were available and were only given information about the one they were randomly assigned. This process was completed between February and July 2014: 1) inclusion criteria: 3^rd^ to 14^th^ of February; 2) Pre-intervention: 17^th^ of February to 30^th^ of April; and 3) Post-Intervention: 1^st^ of May to 18^th^ of July.

### Interventions

**Figure 1:**
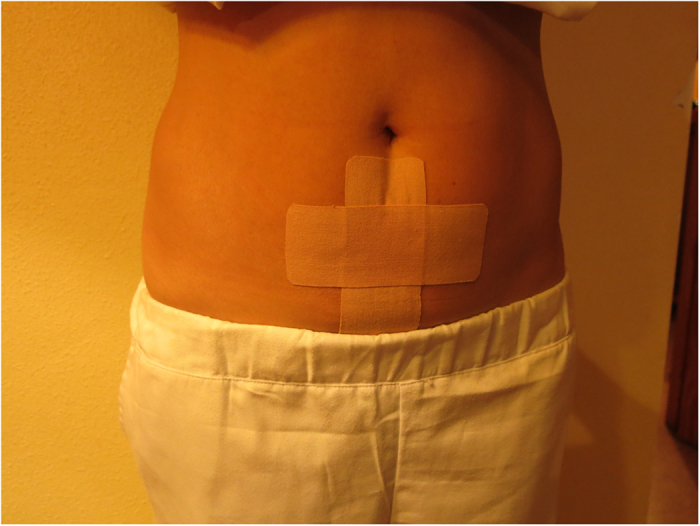
Medical Taping Concept in the abdomen area (intervention group). The copyright holder (ATENA PRODUCTOS FARMACÉUTICOS, S.L.) has approved the utilization of this figure.

**Figure 2:**
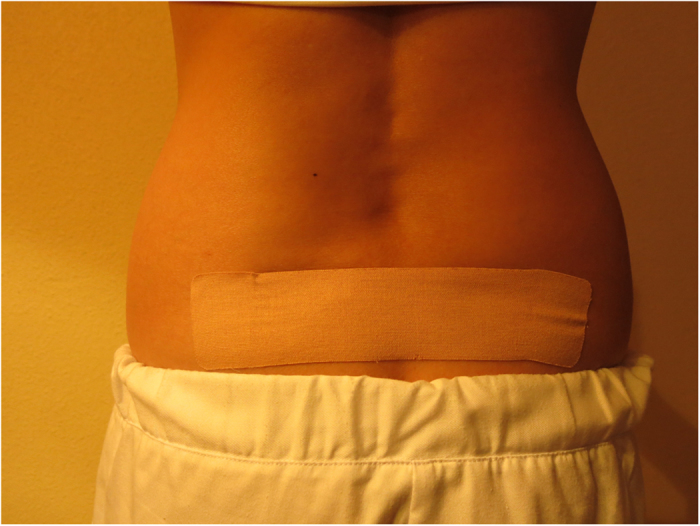
Medical Taping Concept in the low back area (intervention group). The copyright holder (ATENA PRODUCTOS FARMACÉUTICOS, S.L.) has approved the utilization of this figure.

Intervention group (Supplementary Videos S1-3): Whilst the patient is standing –up, 3 bandages of a special elastic and hypoalergenic surgical tape (Cure Tape®) which are all 5 cm wide, are applied to dermatomes 11 and 12: 1^st^) 12 cm long: applied vertically between the belly-button and the pubis (Fig. 1). The central part of the adhesive is removed and is fastened by the lateral anchors. The tension is of 25%, which implies that the tape’s length is increased by 3 cm. When applying the tape, the participant inhales and slightly bends backwards extending the torso. The centre of the tape is adhered and afterwards the rest of the protectors are removed from the lateral anchors (rest of the paper) and the rest of the tape is adhered. 2^nd^) 12 cm length: to be applied horizontally (perpendicular to the previous tape). The same tension is applied and following the same procedure (Fig. 1). 3^rd^) 20 cm long: The participant has to slightly lean forward. She has to remove the central part of the adhesive and hold it by the lateral anchors. The tension is 25%, therefore increasing the length by 5cm. The same procedure as before is followed, except that the tape is to be placed in the lumbar areas, covering both posterior-superior iliac spines (Fig. 2).

**Figure 3:**
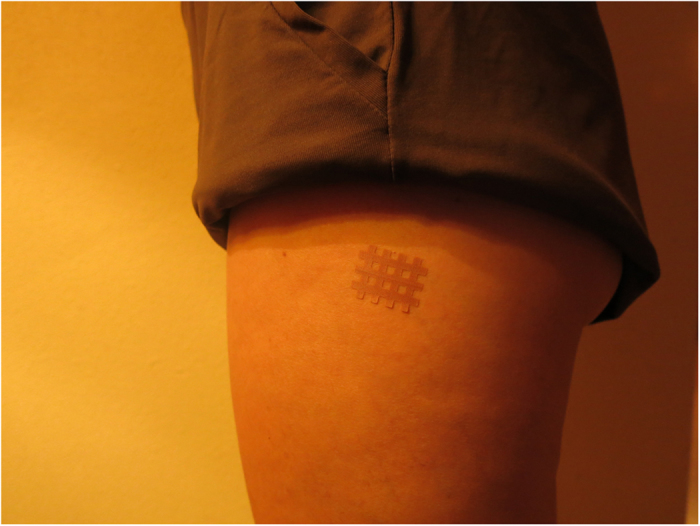
Spiral Tape in the greater trochanter area (control group). The copyright holder (ATENA PRODUCTOS FARMACÉUTICOS, S.L.) has approved the utilization of this figure.

Control group: Two special non-extendible meshed bandage patches measuring 2.5 × 2 cm (Cross Tape®) away from dermatomes 11 and 12. Each patch is applied to the external part of each thigh, at the greater trochanter (Fig. 3 and Supplementary Video S4).

The material for both groups can tolerate being wet once applied and it dries fast and easily, thus avoiding damp patches and skin alterations. This allows the user to wear them for periods of 3 to 5 days with no need to remove them. The materials are to be placed once the menstrual pain begins and are to remain adhered for about 4–5 days until the pain disappears.

As a control we chose to use a technique that was not adhered to the dematomes being studied. Thus, we were able to assess more precisely the benefits of the intervention. As a control group this technique was preferred as opposed to leaving the participants with no physiotherapy treatment. This was because if we did it the other way, it would be very possible that the participants would refuse to participate in a clinical trial about treatment for PD.

### Variables and measurements

Outcomes: Pre-intervention and post-intervention increase in pain difference measured 2 hours after commencement of pain (pain was measured when it commenced and two hours thereafter), difference in number of tablets for menstrual pain (of any type during the whole menstrual cycle) taken post- and pre-intervention, and associated symptoms in post-intervention (fatigue, vomiting, diarrhoea, nausea and others).

The researchers decided to establish a two hour period because it was not considered ethical to forbid the participants from taking pain relief during long periods of time. This also allowed us to assess the effectiveness of MTC in the short term, as in the long term we were able to assess it based on the intake of pills.

Pain was measured in different parts of the body (abdomen, legs, lumbar area and head). Because we were using data from two menstrual cycles before and after the intervention, pain scores and number of tablets were averaged for the two months of each period (pre and post-intervention). Regarding the symptoms in post-intervention, participants were considered to have suffered them if they appeared at least once in the two menstrual cycles. The participants had to answer if the symptoms had appeared, this is to say, this variable was assessed qualitatively (symptom YES, symptom NO).

The instruments used to measure the outcomes were: pain was assessed using a 10 point scale (0 = no pain and 10 = maximum pain)^29^; number of tablets taken and symptoms were assessed using self-reported answers written by the participants.

To check for homogeneity in the groups (intervention and control) during the study, the following variables were assessed: length of the menstrual cycle (21–27, 28–31 and 32–35 days), length of menstruation (1–3, 4–6 and 7–9 days), family history of primary dysmenorrhoea (yes/no), menstrual flow (low, medium and high), natural analgesic actions (yes/no), smoking (yes, no and former smoker), fatigue (yes/no), vomiting (yes/no), diarrhoea (yes/no), nausea (yes/no), other symptoms (yes/no), age (years), body weight (kg) and body height (m).

For both groups the same measurements methods were performed.

### Sample size

Sample size was calculated to determine if there were differences between the two means (t-tests). For this we used the following parameters: Type I error of 5%, 90% power, an expected difference of 0.75, an expected SD of 1.3 and an expected drop out of 40%. The expected difference and the standard deviation were obtained from a pilot study using 18 participants. With all this, the sample size obtained was 162 (81 per each group).

### Statistical methods

The descriptive analysis was carried out using the standard methods in clinical research, using absolute and relative frequencies for qualitative variables, and for quantitative variables means and standard deviations (normal distribution) were utilized, or medians and inter-quartile ranges (no normal distribution). The Z test was used to test whether the same proportion of women were being lost in each group, both during the intervention and during follow up. Homogeneity was tested (intervention and control) for allocation and analysis, and for the participants that abandoned the study. This was done using X^2^ (Pearson or Fisher), t-student, Mann-Whitney U, ANOVA and Kruskal-Wallis tests; depending on the type of variable. With the final sample, differences for the outcomes were analyzed for each group. Finally, we performed the intention to treat to study the dropouts, calculating relative risk (RR), absolute risk reduction (ARR), relative risk reduction (RRR), and number needed to treat (NNT). All analyses were performed using a Type I error of 0.05 and confidence intervals were calculated for each relevant parameter. All analyses were performed using IBM SPSS Statistics 19.0.

### Ethical consideration

All answers were recorded anonymously and individually, with no interaction between the participants. To identify each participant, we used the last 4 digits of their ID cards. If there were any overlaps, numbers _0, _1, etc were added.

The study received ethical approval from the Ethic and Experimental Research Committee from the Miguel Hernández University of Elche (June 6, 2013). Each participant signed a written informed consent. The protocol for this trial has been registered at ClinicalTrials.gov, ID number NCT02114723 (April 7, 2014). This registration was performed after the enrolment process (pre-intervention) but before randomization (post-intervention). The authors confirm that all ongoing and related trials for this drug/intervention are registered.

All experiments were performed in accordance with relevant guidelines and regulations.

## Results

**Figure 4:**
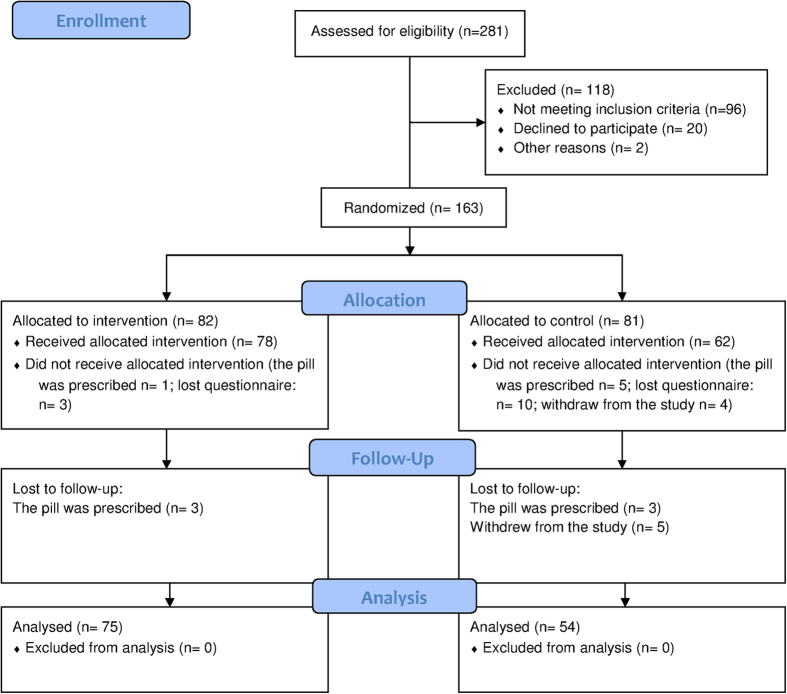
CONSORT flow chart. Women who withdrew from the study was because we could not contact with them again.

Figure 4 shows the flow chart of the study. Out of the 281 possible participants 118 were excluded, thus leaving a sample of 163 women to be randomized into two groups. After allocation, and before explaining the intervention, 4 women of the intervention group and 19 of the control group did not receive the technique. This presented differences between the groups (Z = 3.1817, p = 0.0015). Data was collected for 140 women. Out of these 11 were lost to follow-up (intervention, 3; control, 8). Thus leaving a final sample composed of 75 women in the intervention group and 54 in the control group.

Both groups were similar in the distribution of baseline characteristics (p: 0.141–0.979) (Table 1).

Table 1 shows there were no differences between the women that dropped out from the study during follow-up and those that completed it (p: 0.061–0.99).

Regarding homogeneity between the groups in the final sample, no statistically significant differences were found (p: 0.450–0.987) (Table 1).

Table 2 shows the analyses performed for the outcome measures in the final sample. The differences observed for the outcomes, along with their p-values between the control and intervention group were: difference in number of tablets (intervention: −1.14 ± 1.33; control: −0.47 ± 0.94; p = 0.002), difference in abdominal pain (intervention: −0.34 ± 2.50; control: 0.71 ± 1.82; p = 0.010), difference in low back pain (intervention: −0.46 ± 1.28; control: −0.11 ± 1.62; p = 0.168), difference in headache (intervention: −0.20 ± 1.37; control: −0.11 ± 1.18; p = 0.701) and difference in leg pain (intervention: −0.25 ± 1.31; control: 0.31 ± 1.24; p = 0.015).

As for the post-intervention symptoms, no statistically significant differences were found between the groups (p: 0.430–0.870) (Table 2).

Regarding the intention-to-treat analysis, we obtained the following results in favor of the intervention group: RR, 0.26 (95% CI: 0.12–0.55); RRR, 0.74 (95% CI: 0.45–0.88); ARR, 0.25 (95% CI: 0.13–0.37); NNT, 5 (95% CI: 3–8).

## Discussion

This study provides an analysis of more than 100 women in which we assessed the effectiveness of MTC to reduce the pain caused by PD. This technique managed to reduce pain in a statistically significant manner two hours after it started. According to the scale of pain, there was a decrease of 1 point for abdominal pain and half a point for leg pain. Also, after the first two hours of the pain commencing, we observed a decrease of 0.7 in the number of tablets taken by the intervention group. In addition, no differences were found for the other symptoms we analyzed. Finally, the intervention technique was better accepted in our study, as the drop-out rate was higher in the control group. This was also found in the intention to treat analysis.

In our literature review, to our knowledge there is only one other study in English that assessed a sample of 34 women suffering from PD using Kinesiotaping and Spiral Taping in a three-armed clinical trial. This study assessed mainly the pain and pre-menstrual symptoms in specific areas, and only analyzed menstrual pain globally, that is to say, not in specific areas of the body. Nevertheless, it is difficult to compare our results to this study, as there was no strict control of medication intake during follow-up and this could be affecting their results^25^. In this sense, the results from our study are innovative in the way they assessed the true effectiveness of MTC, as we suggest that pain is reduced if the participant doesn’t take any medication for two hours; and that if after this two hour period medication is used, the number of tablets is cut down.

It has been suggested that transcutaneous electrical stimulation acts upon pain in PD, producing sensory tactile pulses^13^, which are transmitted to higher levels and can block or reduce the arrival of pain sensations to the brain^24^. In a similar manner, when MTC is applied, the psychogenic excitation of the central analgesic system can be engaged when the bandage is in contact with the skin and thus releases sensory tactile pulses that achieve the same effect. Also, MTC could be stimulating the nociceptors, as it is able to lift the skin and therefore allow drainage and decompression of the area.

Our findings show lower pain levels after two hours in the intervention group. To be able to assess if MTC is completely effective, and that no medication is needed after this time, it would be necessary to carry out studies in which this trial is repeated, where either pain medication is completely abolished or homogenizing them to be able to control it. This type of study is very difficult to achieve due to the great variety of treatments used for PD, and it would be ethically incorrect to forbid a participant from using pain relief, therefore a possible solution would be to repeat this study under better conditions with volunteers who are willing to forego medication for four menstrual cycles. On the other hand, we must consider that although the difference between both techniques was statistically significant, this was only represented by a maximum difference of 1 point in the pain scale. In other words, the proposed technique could relieve pain and therefore reduce the use of pharmacological treatments, but it would not be a substitute.

The main strength of this study is that it is the first time that MTC has been assessed for effectiveness on menstrual pain in specific areas and the use of medication in women with PD, therefore our results are innovative.

As for the limitations, it was surprising to find that there were statistically significant differences in group allocations, as at that moment none had been briefed about the use of the techniques. Nevertheless, once the participants had been briefed on the use of the techniques, the homogeneity of the groups remained for the rest of the study. Finally, we have to consider that our trial was not blind, as it was the participants who were themselves taking their own measurements and they knew what group they had been assigned to. Nevertheless, we have to take into account that pain is always self-reported and that because the techniques were so different, it was impossible to assure the participants remain blind.

## Conclusion

MTC seems to be a complementary effective non-pharmacological treatment, which is simple, comfortable and self-applicable in PD. This technique reduced abdominal and leg pain when the participant was not taking medication (two hours after start of pain) and medication intake was also reduced (between two hours and end of menstrual cycle). Nevertheless, we must take into account that the drop-out rate was lower in the intervention group, therefore if our results are repeated in other studies where the rate is similar between groups (control and intervention) and these obtain efficient results on behalf of the MTC during the duration of the menstrual period and remove the need for medication, we could be availing of a complementary therapy to pharmacological treatments for PD.

## Additional Information

**How to cite this article**: Tomás-Rodríguez, M. I. *et al*. Effectiveness of medical taping concept in primary dysmenorrhoea: a two-armed randomized trial. *Sci. Rep.* **5**, 16671; doi: 10.1038/srep16671 (2015).

## Supplementary Material

Supplementary Information

Supplementary Video 1

Supplementary Video 2

Supplementary Video 3

Supplementary Video 4

## Figures and Tables

**Table 1:** Descriptive analysis for women who participated in the study in a Spanish region, 2014 data.

Variable	Allocation*			Follow-up*^,†^			Analysis*		P
	Intervention group	Control group	P	Withdrew from the study	Completed the study		Intervention group	Control group	
	n = 78	n = 62		n = 11	n = 129	P	n = 75	n = 54	
Length of the menstrual cycle:			0.574			0.086			0.811
21–27 days	15(20.5)	17(27.9)		5(50.0)	27(21.8)		14(20.0)	13(24.1)	
28–31 days	43(58.9)	34(55.7)		3(30.0)	74(59.7)		42(60.0)	32(59.3)	
32–35 days	15(20.5)	10(16.4)		2(20.0)	23(18.5)		14(20.0)	9(16.7)	
Length of menstruation:			0.939			0.098			0.791
1–3 days	6(7.9)	5(8.1)		2(18.2)	9(7.1)		6(8.2)	3(5.6)	
4–6 days	65(85.5)	52(83.9)		7(63.6)	110(86.6)		63(86.3)	47(87.0)	
7–9 days	5(6.6)	5(8.1)		2(18.2)	8(6.3)		4(5.5)	4(7.4)	
Family history of PD	64(82.1)	49(79.0)	0.653	9(81.8)	104(80.6)	>0.99	61(81.3)	43(79.6)	0.809
Menstrual flow:			0.618			>0.99			0.720
Low	7(9.0)	3(4.8)		0(0.0)	10(7.8)		7(9.3)	3(5.6)	
Medium	49(62.8)	42(67.7)		8(72.7)	83(64.3)		47(62.7)	36(66.7)	
High	22(28.2)	17(27.4)		3(27.3)	36(27.9)		21(28.0)	15(27.8)	
Natural analgesic actions	35(44.9)	24(38.7)	0.463	5(45.5)	54(41.9)	>0.99	33(44.0)	21(38.9)	0.562
Smoking:			0.220			0.244			0.450
Yes	8(10.3)	6(9.7)		0(0.0)	14(10.9)		8(10.7)	6(11.1)	
No	66(84.6)	56(90.3)		10(90.9)	112(86.8)		64(85.3)	48(88.9)	
Former smoker	4(5.1)	0(0.0)		1(9.1)	3(2.3)		3(4.0)	0(0.0)	
Fatigue	55(70.5)	43(69.4)	0.882	7(63.6)	91(70.5)	0.734	53(70.7)	38(70.4)	0.971
Vomit	6(7.7)	2(3.2)	0.301	0(0.0)	8(6.2)	>0.99	6(8.0)	2(3.7)	0.467
Diarrhoea	25(32.1)	20(32.3)	0.979	3(27.3)	42(32.6)	>0.99	24(32.0)	18(33.3)	0.873
Nausea	23(29.5)	17(27.4)	0.788	4(36.4)	36(27.9)	0.510	22(29.3)	14(25.9)	0.670
Other symptoms	15(19.2)	11(17.7)	0.822	2(18.2)	24(18.6)	>0.99	15(20.0)	9(16.7)	0.631
Age (years)	20.6 ± 2.5	20.4 ± 2.3	0.668	20(20–22)	20(19–22)	0.273	20.5 ± 2.3	20.3 ± 2.1	0.537
Body weight (kg)	58.2 ± 6.4	60.1 ± 8.1	0.141	62.5 ± 9.7	58.7 ± 6.9	0.099	58.4 ± 6.2	59.3 ± 7.9	0.467
Body height (m)	1.6 ± 5.6	1.7 ± 6.1	0.502	1.7(1.6–1.7)	1.7(1.6–1.7)	0.222	1.7 ± 5.4	1.7 ± 6.3	0.987

PD, primary dysmenorrhoea. *quantitative variables are described using mean ± standard deviation or median (interquartile range) for non-normal distributions with <50 data in the group, and qualitative variables using absolute and relative frequencies. ^†^Withdrew from the study: intervention group, 3 (27.3); control group, 8 (72.7); Completed the study: intervention group, 75 (58.1); control group, 54 (41.9). p-value = 0.061.

**Table 2:** Effectiveness of Medical Taping Concept in Primary Dysmenorrhoea in women from a Spanish region, 2014 data.

Variable	Intervention group	Control group	Difference (means/proportions)	p-value
	n=75	n=54	(95% CI)	
Difference in number of tablets	−1.14 ± 1.33	−0.47 ± 0.94	−0.67 (−1.09, −0.25)	0.002
Difference in abdominal pain	−0.34 ± 2.50	0.71 ± 1.82	−1.05 (−1.84, −0.26)	0.010
Difference in low back pain	−0.46 ± 1.28	−0.11 ± 1.62	−0.35 (−0.86, 0.15)	0.168
Difference in headache	−0.20 ± 1.37	−0.11 ± 1.18	−0.09 (−0.55, 0.37)	0.701
Difference in leg pain	−0.25 ± 1.31	0.31 ± 1.24	−0.56 (−1.01, −0.11)	0.015
Fatigue	35(46.7)	29(53.7)	−0.07 (−0.26, 0.12)	0.430
Vomit	3(4.0)	4(7.4)	−3.4 (−0.13, 0.07)	0.451
Diarrhoea	16(21.3)	13(24.1)	−2.7 (−0.19, 0.14)	0.713
Nausea	16(21.3)	13(24.1)	−2.7 (−0.19, 0.14)	0.713
Other symptoms	9(12.0)	7(13.0)	−1.0 (−0.14, 0.12)	0.870

Quantitative variables are described using mean ± standard deviation and qualitative variables using absolute and relative frequencies.

The pain was assessed using a scoring system (0–10).

The symptoms were assessed in the post-intervention period.
