# Supplementary material for: Effectiveness of medical taping concept in primary dysmenorrhoea: a two-armed randomized trial
Source: Sci Rep. 2015 Nov 13;5:16671. doi: 10.1038/srep16671 (PMC4643292; doi:10.1038/srep16671)
Supplement: Supplementary Information [file srep16671-s1.doc]

Title: Effectiveness of medical taping concept in primary dysmenorrhoea: a two-armed randomized trial.

Authors: María Isabel Tomás-Rodríguez, Antonio Palazón-Bru, Damian Robert James Martínez-St. John, José Vicente Toledo-Marhuenda, María del Rosario García-Asensio, Vicente Francisco Gil-Guillén.

Descriptions of the supplementary videos:

Supplementary Video 1: How to prepare the bandages (intervention).

The copyright holder (ATENA PRODUCTOS FARMACÉUTICOS, S.L.) has approved the utilization of this video.

Supplementary Video 2: How to use the Medical Taping Concept in the abdomen area (intervention group).

The copyright holder (ATENA PRODUCTOS FARMACÉUTICOS, S.L.) has approved the utilization of this video.

Supplementary Video 3: How to use the Medical Taping Concept in the low back area (intervention group).

The copyright holder (ATENA PRODUCTOS FARMACÉUTICOS, S.L.) has approved the utilization of this video.

Supplementary Video 4: How to use the Spiral Tape (control group).

The copyright holder (ATENA PRODUCTOS FARMACÉUTICOS, S.L.) has approved the utilization of this video.
